# An inverse method for mechanical characterization of heterogeneous diseased arteries using intravascular imaging

**DOI:** 10.1038/s41598-021-01874-3

**Published:** 2021-11-18

**Authors:** Bharath Narayanan, Max L. Olender, David Marlevi, Elazer R. Edelman, Farhad R. Nezami

**Affiliations:** 1 Institute for Medical Engineering and Science, Massachusetts Institute of Technology, Cambridge, MA 02139 USA; 2 Institute of Chemical Sciences and Engineering, École Polytechnique Fédérale de Lausanne, 1015 Lausanne, Vaud Switzerland; 3 Department of Mechanical Engineering, Massachusetts Institute of Technology, Cambridge, MA 02139 USA; 4 Cardiovascular Division, Brigham and Women’s Hospital, Harvard Medical School, Boston, MA 02115 USA; 5 Thoracic and Cardiac Surgery Division, Brigham and Women’s Hospital, Harvard Medical School, Boston, MA 02115 USA

**Keywords:** Biomedical engineering, Interventional cardiology

## Abstract

The increasing prevalence of finite element (FE) simulations in the study of atherosclerosis has spawned numerous inverse FE methods for the mechanical characterization of diseased tissue in vivo. Current approaches are however limited to either homogenized or simplified material representations. This paper presents a novel method to account for tissue heterogeneity and material nonlinearity in the recovery of constitutive behavior using imaging data acquired at differing intravascular pressures by incorporating interfaces between various intra-plaque tissue types into the objective function definition. Method verification was performed in silico by recovering assigned material parameters from a pair of vessel geometries: one derived from coronary optical coherence tomography (OCT); one generated from in silico-based simulation. In repeated tests, the method consistently recovered 4 linear elastic (0.1 ± 0.1% error) and 8 nonlinear hyperelastic (3.3 ± 3.0% error) material parameters. Method robustness was also highlighted in noise sensitivity analysis, where linear elastic parameters were recovered with average errors of 1.3 ± 1.6% and 8.3 ± 10.5%, at 5% and 20% noise, respectively. Reproducibility was substantiated through the recovery of 9 material parameters in two more models, with mean errors of 3.0 ± 4.7%. The results highlight the potential of this new approach, enabling high-fidelity material parameter recovery for use in complex cardiovascular computational studies.

## Introduction

The ubiquity of clinical imaging and computational resources have brought patient-specific computational studies to the forefront of cardiovascular research and development^1,2^. Structural simulations using finite element (FE) modeling have become commonplace in studying vascular interventions and devices (e.g. stents), providing insights into the impact of wall heterogeneity on interventional procedures^3–5^. They have similarly been used to study the distribution of plaque structural stresses (PSS), a well-known proxy for predicting plaque rupture^6–8^. Likewise, fluid–structure interaction (FSI) simulations have been used to ascertain the impact of plaque heterogeneity on atherosclerotic growth^9^. In all cases, such high-fidelity atherosclerotic vascular modeling is predicated on the availability of detailed in vivo morphology of tissue components and their physiologically adherent constitutive material properties.

The advent of high-resolution in vivo imaging techniques has enabled great progress in determining tissue morphology, enabling the characterization of atherosclerotic tissue in its native state through virtual histology (VH) assessment. The most clinically prevalent of these is VH-intravascular ultrasound (IVUS), which automatically classifies vascular tissue through spectral decomposition of the acoustic acquisition signal^10–12^. Recently, alternative classification schemes have been developed, incorporating machine learning methods to perform similar segmentation using IVUS or optical coherence tomography (OCT) images^13–15^. Such classified images can then be used to build patient-specific FE models^16,17^.

The determination of physiologically representative material properties remains a comparably challenging task. A number of studies have used ex vivo testing methods to fit observed tissue behavior to material models of differing complexities^18–20^. It is tempting to directly utilize the parameters reported by such studies in a lesion-specific simulation setting. However, such generalizability is precluded by the great variance in atherosclerotic mechanical properties between and within patients^19,21^, which directly impact the stresses predicted by subsequent FE simulations^22^. In addition, ex vivo testing can alter the properties of arterial tissue by removing it from its natural environment, rendering the physiological relevance of such material characterization uncertain. Hence, accurate prediction of the patient-specific mechanical response of atherosclerotic tissue mandates subject-specific in vivo tissue material properties.

FE modeling itself provides a possible mechanism by which to determine such patient-specific in vivo tissue material properties through inverse FE methods. While forward FE modeling recovers displacements from known material properties and loading conditions, inverse FE modeling recovers material properties from known displacements and loading conditions. These methods utilize iterative rounds of FE simulations, where local material parameters are continuously tuned to minimize a predefined objective function to replicate experimentally measured displacements. Early on, two-dimensional (2D) inverse studies of diseased arteries recovered single-parameter constitutive behavior of atherosclerotic tissue, matching displacement fields presumed available through elastographic imaging^23–26^. These pioneering studies were limited by their inability to capture the complex multidimensional linked stress–strain relationships in the cardiovascular system due to the use of single parameter constitutive models^27^. High-fidelity computational studies tend to instead approximate the behavior of tissue components through multi-parameter models, such as the Gasser–Ogden–Holzapfel (GOH), Mooney-Rivlin, and Yeoh^27,28^. In addition, the 2D nature of these works necessarily could not incorporate out-of-plane stresses, neglecting effects of physiologic undulations of the vessel wall and the 3D motion of the heart itself.

Single-parameter models were employed to avoid over-parameterization and ensure solution uniqueness when using displacement data derived from images obtained at two distinct intravascular pressures^25,26,29^. To enable recovery of higher-parameter material models, one could instead acquire data at several intravascular pressures, as previously done in 2D^29^ and 3D^30^. However, increasing the number of required image limits clinical translation. An alternative approach is to incorporate a greater degree of information by aiming to match the entire inner and outer surfaces through inverse methods, as done by Liu et al*.*, who recovered a 5-parameter GOH model for homogeneous aortic tissue^28^. However, their work utilized computed tomography scans lacking heterogeneous morphological information, rendering multi-material, heterogeneous tissue characterization infeasible.

Existing approaches to in vivo tissue characterization have thus compromised on either the number of tissue components or the model complexity. Those that strove to combine both relied upon imaging information from several image acquisitions^29^, severely hindering clinical translation. The current study improves upon these systems by describing a robust inverse FE method that can obtain multiple patient-specific material parameters for several 3D lesion components using only two sets of in vivo image acquisitions. This multi-parameter recovery is facilitated by the incorporation of micro-morphological information in the form of intra-plaque interfaces into the objective function—a development made possible by the high-resolution intravascular imaging of OCT or IVUS. The presented method for multi-parameter, multi-component material characterization ultimately paves the way for patient-specific clinical interventions that take into account the unique mechanistic state that each diseased vessel presents.

## Methodological framework

**Figure 1:**
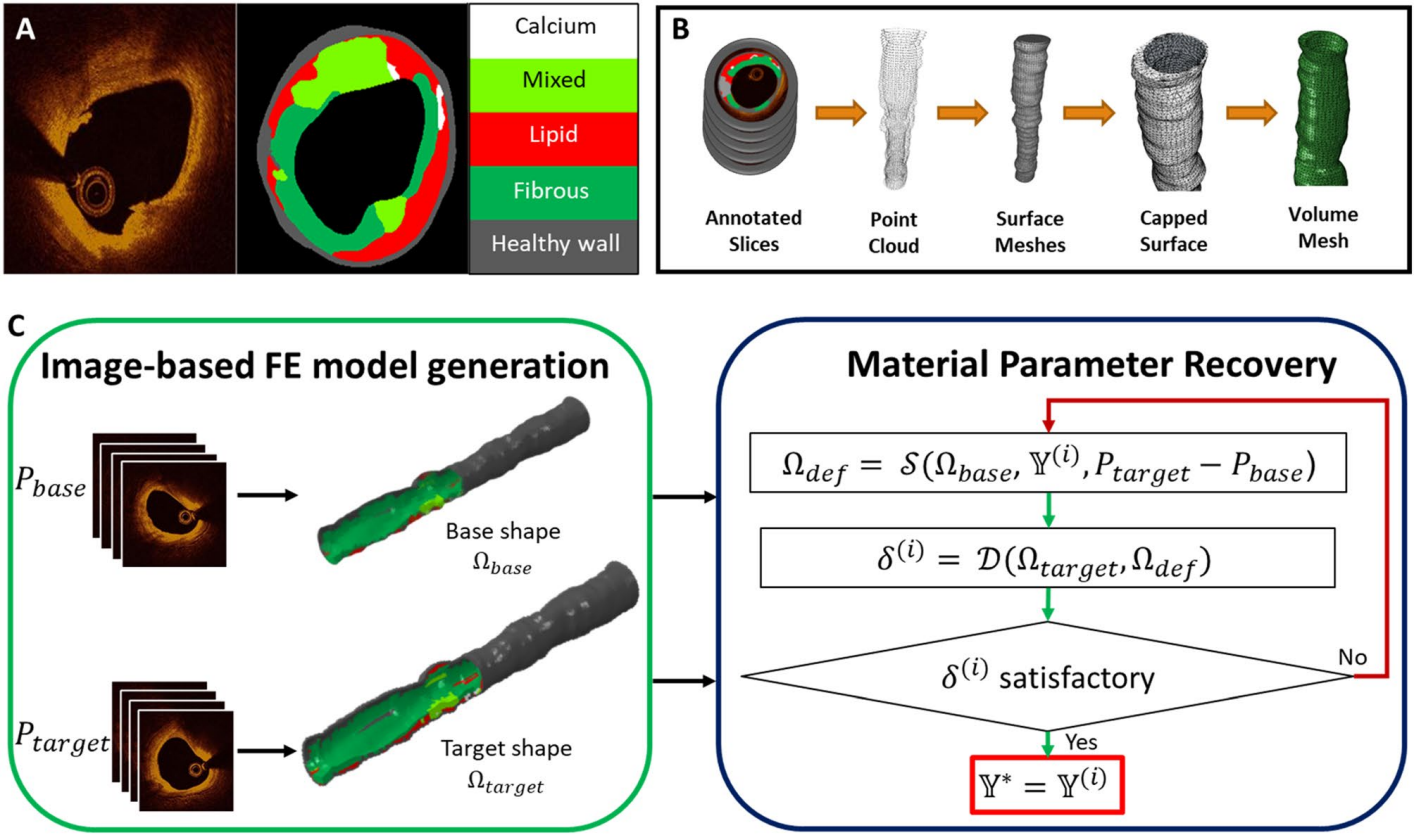
(**A**) From a raw OCT image *(left)*, a morphological map *(middle)* was generated by an automated annotation algorithm. Here, different tissue types are represented by different colors *(right)*. (**B**) To generate a volume mesh from a set of annotated slices, the slices were first converted to a point cloud, which was subsequently used to generate an open surface mesh. After closing the open surface mesh, the closed surface mesh was used as a scaffold to generate a 3D volume mesh. Material classes were subsequently assigned to elements on the basis of the morphological annotations (as seen in **C**). (**C**) The inverse FE method recovered the material parameters of multiple plaque components from two different input image sets. Workflows illustrated in panels A and B were used to convert OCT images from pullbacks acquired at two different pressures, P_base_ and P_target_, into 3D FE meshes, $\Omega_{\text{base}}$ and ${\Omega_{\text{target}}}$, with heterogeneous material distributions (left box). A method was developed to subsequently utilize these models to recover the vector of material properties ($\mathbb{Y}$*) that resulted in the observed displacements between them (right box). In each iteration of the method, FE simulation ($\boldsymbol{\mathcal{S}}$) of the deformed shape (${\Omega_{\text{def}}}$) was performed by applying the pressure differential P_target_ − P_base_ to the base shape using the current material parameter vector ($\mathbb{Y}$^(i)^). The quantitative difference (*δ*^(i)^) between ${\Omega_{{\text{def}}}}$ and the target shape (${\Omega_{{\text{target}}}}$) was then computed by difference operator ($\boldsymbol{\mathcal{D}}$) and used to refine the subsequent estimate of material parameters, $\mathbb{Y}$^(i+1)^.

We present a method to recover the material properties of multiple arterial plaque components using two sets of intravascular imaging data acquired at different intraluminal pressures (Fig. 1). A pre-processing step was conducted to first convert in vivo images into 3D FE geometries with heterogeneous material attribution. These geometries were then utilized by the presented inverse method to recover the material properties that reproduce the behavior manifested by the two imaged states.

## Image-based FE model generation

The FE model generation protocol accepted as input OCT image data acquired in the course of clinical care. Given these data, the inner and outer borders of the vessel wall were identified and fit with a smooth, continuous surface in 3D^31^. The resulting region of interest was then characterized with a validated deep learning method for classifying tissue micromorphology in OCT images^13^, which automatically annotated frames with the spatial distribution of non-pathological and diseased (calcified, lipid, fibrotic, or mixed) tissue. A subset of the total acquisition length was taken to exclude low-quality images and reduce computational costs. The final output of these steps was a point cloud set consisting of pixel coordinates and corresponding tissue labels from the selected segment.

From the point cloud data, a volumetric FE model was then generated. The points representing the inner and outer surfaces of the imaged vessel were converted into triangulated meshes by Poisson surface reconstruction, then connected at each end to form a closed surface mesh. This resultant mesh was converted into a volume mesh of tetrahedral elements. Each element in the mesh was then assigned to the material class associated with the point in the annotated OCT dataset closest to its centroid. The resulting 3D tetrahedral mesh (Ω)—with heterogeneous material distribution—represents the transformation of visually-encoded data captured during clinical imaging into a discretized volumetric model amenable to structural simulation.

## Inverse FE method

The goal of the inverse FE method was to find the vector of optimal material parameters, ${{\mathbb{Y}}^*}$, such that the base and target geometries, ${\Omega_{{\text{base}}}}$ and ${\Omega_{{\text{target}}}}$, are matched under the given loading condition, ΔP. In intravascular simulations, ΔP = P_target_ − P_base_ where P_base_ and P_target_ represent the different intraluminal pressure states at which the images corresponding to ${\Omega_{{\text{base}}}}$ and ${\Omega_{{\text{target}}}}$ were acquired. Each iteration of the optimization routine utilized an FE simulation to calculate the deformed geometry ${\Omega_{{\text{def}}}}$ whose deviation from ${\Omega_{{\text{target}}}}$ was quantified by the operator D and represented by the objective function ${\delta^i} = \boldsymbol{\mathcal{D}}\left( {{\Omega_{{\text{def}}}},{\Omega_{{\text{target}}}}} \right)$.

### Definition of the objective function

**Figure 2:**
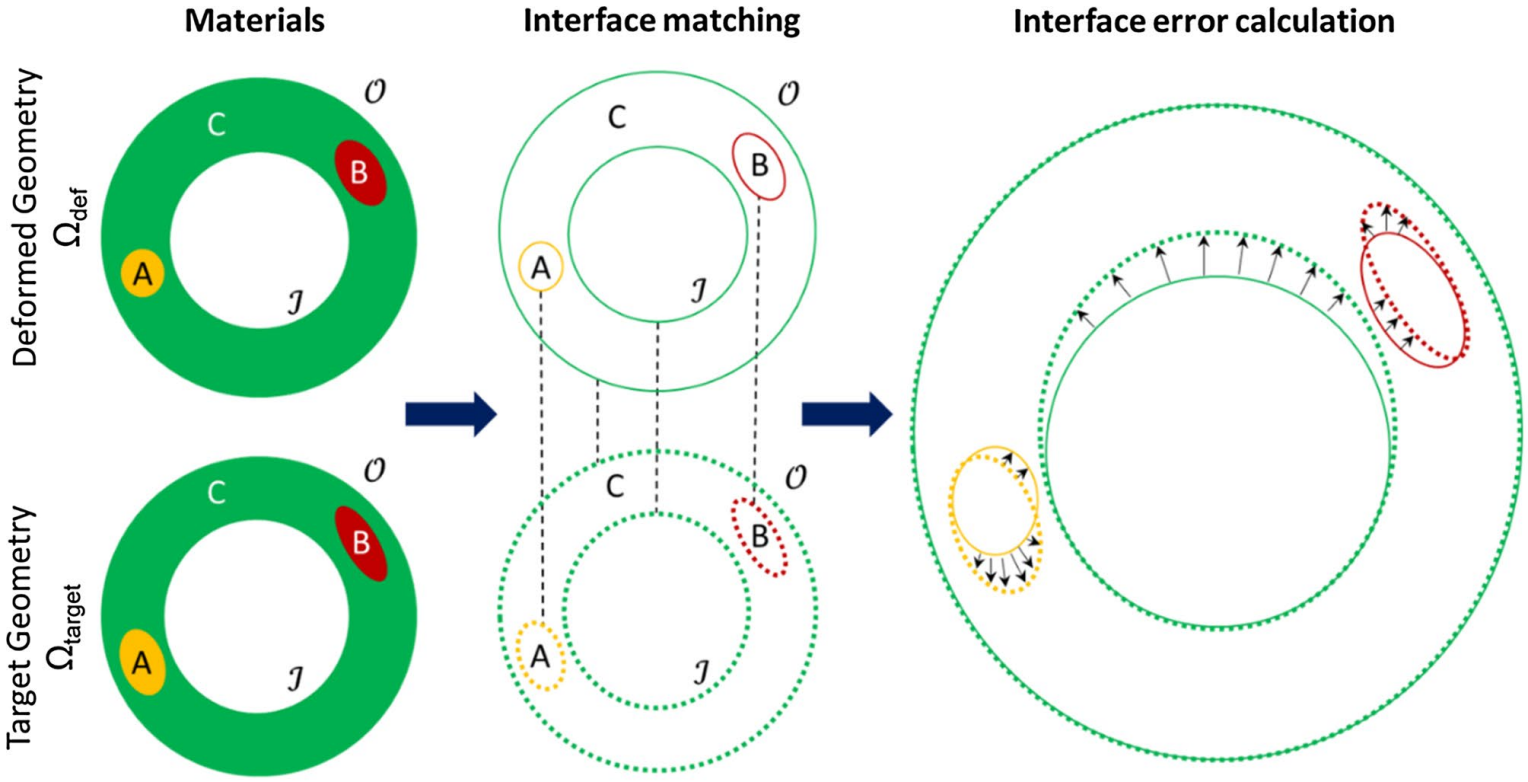
Schematic representation of a difference operator quantifying multiple feature changes. ${{\Omega }_{{\text{def}}}}$ and ${{\Omega }_{{\text{target}}}}$ have five node sets contained in list ${\boldsymbol{\mathcal{N}}}$: three represent material regions (**A**, **B**, and **C**), and the other two include the inner and outer surfaces (${\mathscr{J}}$ and ${\mathscr{O}}$). Corresponding interfaces on the deformed (solid) and target (dashed) geometries were compared by calculating the average Euclidean distance between their nodes (black arrows), based on a nearest neighbor search.

There are various ways to define the objective function. One possibility is to compare macro-morphological entities, which in the case of vascular modeling could be vessel diameters/circumferences or the inner and outer surfaces of the two matched geometries. However, using such ensemble observations can preclude the presence of a single unique solution when trying to recover multiple material parameters^26^. To circumvent this limitation, micro-morphological information in the form of interfaces between tissue regions was also incorporated in the formulation of the objective function. With node sets (contained in an array $\boldsymbol{\mathcal{N}}$) being defined for each material region and for the inner and outer surfaces, an interface was defined as the set of common nodes between two node sets. For each node $r_j^{{\text{target}}|X,Y}$ in the target geometry belonging to an interface between node sets *X* and *Y* (X ∩ Y, ${\mathbf{X}}, {\mathbf{Y}} \in \boldsymbol{\mathcal{N}}:Y \ne X$), the Euclidean distance to the nearest node $r_k^{{\text{def}}|X,Y}$ belonging to the corresponding interface in the deformed base geometry was calculated. The average of all such distances comprised the interface error, ${\epsilon^{X,Y}}$:

1 $${\epsilon^{X,Y}} = \frac{1}{{{N^{{\text{target}}|X,Y}}}}\mathop \sum \limits_{j = 1}^{{N^{{\text{target}}|X,Y}}} \mathop {\min }\limits_{k \in \left\{ {1,...,{N^{{\text{def}}|X,Y}}} \right\}} \left| {r_j^{{\text{target}}|X,Y} - r_k^{{\text{def}}|X,Y}} \right|,$$

where ${N^{{\text{def}}|X,Y}}$ and ${N^{{\text{target}}|X,Y}}$ are the number of nodes in the interfaces of the deformed and target geometries, respectively. To illustrate this formulation, Fig. 2 shows a simplified scenario with multiple plaque components where $\boldsymbol{\mathcal{N}}$  = {A, B, C, ${\mathscr{J}}$, ${\mathscr{O}}$}, yielding four interfaces in total: two between the material region C and material regions A and B (A ∩ C and B ∩ C), and two between the material region C and the inner and outer surfaces (${\mathscr{J}}$ ∩ C and ${\mathscr{O}}$ ∩ C). In this example, there are consequently four interface errors to be calculated: ${\epsilon^{A,C}}$, ${\epsilon^{B,C}}$, ${\epsilon^{\boldsymbol{\mathcal{O}},C}}$ and ${\epsilon^{\mathcal{I},C}}$.

The objective function quantifying the discrepancy between the two geometries at each iteration, *i*, was then formulated as a function of these individual interface errors. In a multi-objective optimization algorithm, the objective function was defined as the vector of all the unweighted interface errors:

2 $$\delta_{MO}^i = \left\{ {{\epsilon^{X,Y}}\forall X,Y \in \boldsymbol{\mathcal{N}}:Y \ne X} \right\},$$

where $\delta_{MO}^i$ is the multi-objective vector. In a single-objective optimization, the objective, $\delta_{SO}^i$, must be a single scalar value and was thus defined as the sum of all interface errors:

3 $$\delta_{SO}^i = \sum {{\epsilon^{X,Y}};\;\;X,Y \in \boldsymbol{\mathcal{N}}:Y \ne X} .$$

This objective function, through the matching of interfaces, allows for the recovery of material parameters for multiple different material sets, using acquisitions at only two states.

### Multi-step optimization process

The solution space for the inverse problem can be highly nonlinear and non-convex. As such, there can exist multiple local minima to which an optimizer may converge, resulting in erroneous recovery of material parameters. To overcome this inherent challenge and increase the probability of attaining a global minimum, a multi-objective genetic algorithm called the Non-dominated Sorting Genetic Algorithm (NSGA-II)^32^ was first used for identifying the global region within which the optimal material parameter set was likely to exist. In NSGA-II, an initial population of parameters was generated using the space-filling Latin Hypercube Sampling method^33^. This population was then propagated over several generations, with the fittest individuals being chosen to stochastically exchange parameters with each other. As it is a multi-objective optimization algorithm, the fitness was measured using the vector of all interface errors $\delta_{MO}^i$ (Eq. 2) and the algorithm converged towards a Pareto optimal set of solutions, defined as a set within which one cannot improve in one dimension of the vector $\delta_{MO}^i$ without sacrificing progress in another dimension.

Once the narrowed region of interest was identified by the NSGA-II algorithm, an evolved single objective, sequential quadratic programming algorithm called NLPQLP^34^ was employed. The individual yielding the minimum sum of all the interface errors ($\delta_{SO}^i$, Eq. 3) across all generations of the NSGA-II run was chosen as the starting parameter vector. The algorithm uses forward finite differences to evaluate the gradient at a given point; if there are *n* parameters to be optimized, each function evaluation requires *n* + 1 simulations, one for the point itself and *n* simulations for the n-dimensional gradient evaluation. The algorithm stops when the difference between successive objectives drops below a given threshold or once it reaches a predefined limit on number of function evaluations.

The stochastic nature of NSGA-II decreased the chances of getting stuck in a local optimum, but limited precision and yielded varying solutions in repeated implementations. In contrast, the NLPQLP algorithm’s deterministic approach guaranteed repeated convergence to an optimal value while being susceptible to convergence to a local, but not global, minimum. The serial combination of the two melded their advantages while counteracting their disadvantages.

### In silico verification of the inverse method

The inverse FE method was verified in silico by recovering mechanical constitutive properties of a patient-specific model generated from clinical images. Noise sensitivity analysis was also carried out on the same patient-specific model to ascertain robustness of the inverse FE framework. Finally, the method was applied to different patient models to assess its generalizability across a range of clinically-relevant diseased vessel phenotypes. All procedures were performed on a CPU with 6 cores running at 2.8 GHz and with 24 GB RAM.

## Data acquisition

OCT image sequences were acquired in three patients during the course of clinical care for the monitoring of coronary artery disease. Patients presented with stable angina or non-ST-elevation myocardial infarction—a common form of acute coronary syndrome, or heart attack—attributed to target lesions in the left anterior descending or left circumflex artery. All data were collected with institutional oversight and informed patient consent; data collection was performed in accordance with relevant guidelines and regulations, and use of images was approved by the IDIVAL Clinical Research Ethics Committee (Santander, Spain). The details of image data acquisition and selection have been previously reported^13,31^. In brief, the data were obtained using an optical frequency domain intravascular imaging system and catheter. The pullbacks were performed at a speed of 20 mm/s, with frames being captured every 0.2 mm over a total length of 54 mm.

## In silico generation of target geometries for method verification

**Figure 3:**
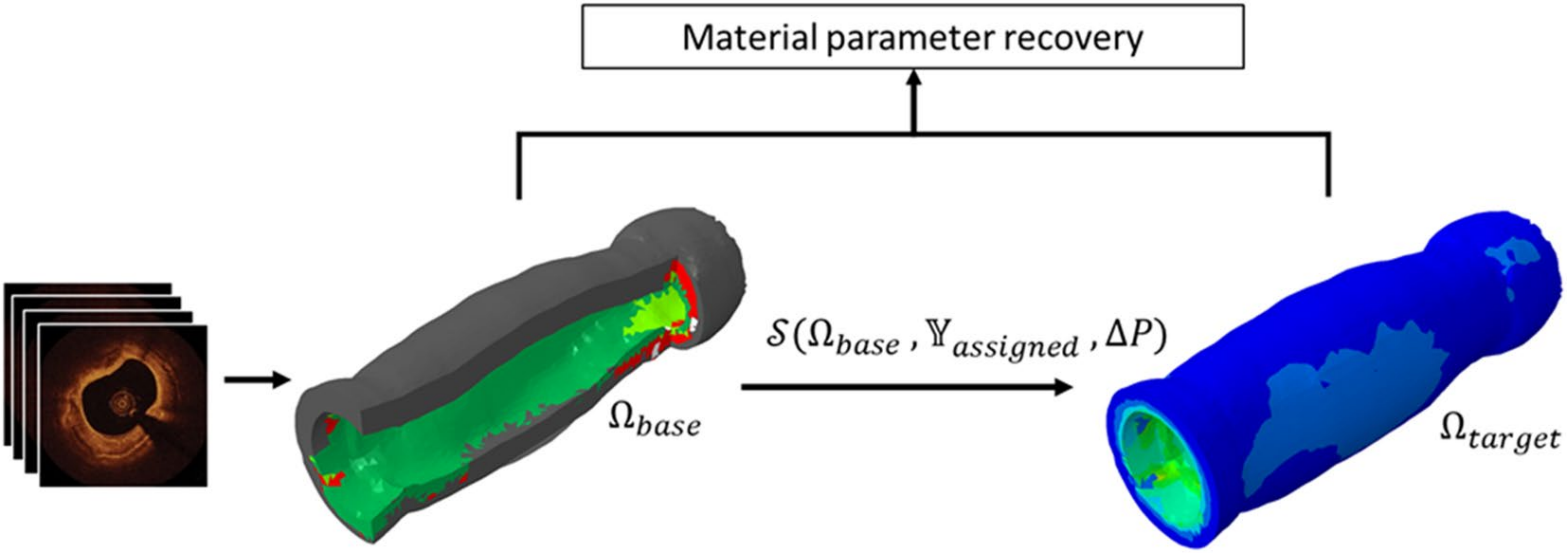
Verification of material recovery using target geometries generated through computational simulations. The imaging data were first converted into a patient-specific FE mesh (${{\Omega }_{{\text{base}}}}$) with material heterogeneity. Material properties (comprising vectors ${{\mathbb{Y}}_{{\text{assigned}}}}$) were then assigned to each tissue class. Pressure (ΔP) was applied to the lumen and the displacements calculated via FE simulation ($\boldsymbol{\mathcal{S}}$). The resultant simulated geometries (${{\Omega }_{{\text{target}}}}$) were formed by adding the calculated, spatially varying displacements at each node to the original nodal coordinates. These virtually generated target geometries were then used together with the patient-specific base geometry (${{\Omega }_{{\text{base}}}}$) to recover the assigned material property vectors.

Using the image-based FE model generation methodology described earlier, the characterized OCT images from a single patient were transformed into a 3D FE mesh with five material regions: fibrous, lipid, calcium, mixed, and healthy wall tissue. As indicated earlier, the presented inverse method assumes the availability of two sets of pullback data with known acquisition pressures. However, common clinical practice and guidelines directs acquisition of only a single image sequence with no corresponding lumen pressure recording. Furthermore, excised tissue was not available from the imaged patients for mechanical testing. Hence, for the sake of verification, the geometries corresponding to the target pullback datasets were generated in silico for assumed material properties (Fig. 3).

The target geometry (${\Omega_{{\text{target}}}}$), which would in practice be derived from a second image acquisition sequence, was instead simulated here in the absence of such repeated acquisition. With the image-derived geometry serving as the base geometry (${\Omega_{{\text{base}}}}$), ${\Omega_{{\text{target}}}}$ was numerically simulated by applying a lumen pressure differential (ΔP) of 60 mmHg to ${\Omega_{{\text{base}}}}$ with both ends constrained in the longitudinal direction:

4 $${\Omega_{{\text{target}}}} = \boldsymbol{\mathcal{S}}\left( {{\Omega_{{\text{base}}}},\Delta P,{{\mathbb{Y}}_{{\text{assigned}}}}} \right),$$

where the operator ${\boldsymbol{\mathcal{S}}}$ represents the forward FE simulation, and ${{\mathbb{Y}}_{{\text{assigned}}}}$ is the vector of assigned material parameters. This pressure differential corresponds to the high end of physiological pulse pressure, the difference between systolic and diastolic blood pressures^35,36^, under the presumption that in clinical practice, the two acquisitions lie within the normal blood pressure range. Verification testing was carried out for two constitutive models of increasing complexity. As a preliminary study, each tissue type was modeled as a linear elastic incompressible material for which stress (σ) is related to infinitesimal strain (ε) by Hooke’s law ($\sigma = E\epsilon$) under the condition that the displacement gradient $\left| {\nabla u} \right| \ll 1$. To demonstrate the multi-parameter recovery capabilities of the inverse method, verification was also undertaken using the 3-parameter nonlinear Yeoh material model, since such models have been shown to be capable of accurately modeling the behavior of soft tissue^27^. Its strain energy (W) function is defined as a polynomial function of the first deviatoric strain tensor, ${I_1} = {\sigma_1} + {\sigma_2} + {\sigma_3}$, where ${\sigma_1},{\sigma_2},{\text{\;and\;}}{\sigma_3}$ are the principal stresses:

5 $$W = {C_{10}}\left( {{I_1} - 3} \right) + {C_{20}}{\left( {{I_1} - 3} \right)^2} + {C_{30}}{\left( {{I_1} - 3} \right)^3},$$

${C_{10}},\;{C_{20}},\;{\text{and}}\;{C_{30}}$ are material constants to be fitted. ${C_{10}}$ can be related to the initial shear modulus such that ${\mu_0} = 2{C_{10}}$ under the assumption of small strains.

Vectors of assigned material parameters (${{\mathbb{Y}}_{{\text{assigned}}}}$) were populated with values established by previous studies of atherosclerotic tissue (Table 1). Published linear elastic moduli values were used as a basis for assigning the stiffness of the arterial wall^19^, lipid pool^37^, and fibrous tissue^19,37,38^. For the Yeoh material model, parameter values for these tissue types were assigned as in a previous study^27^. For both cases, mixed tissue was assigned a value between that of the fibrous and lipid tissue, and calcium was assigned to be much stiffer than the other materials—an approach taken in previous studies as well^30,39^.

**Table 1:** Tissue prevalence, ground truth material parameters, and search bounds used for the patient-specific optimization routine in recovering linear elastic and Yeoh material parameters.

Tissue type	Vessel model volume (%)	Linear elastic parameters		Yeoh material parameters				
		E (kPa)		C_10_ (kPa)		C_20_ (kPa)		C_30_ (kPa)
		Assigned	Search range	Assigned	Search range	Assigned	Search range	Assigned
Artery	64.6	300	100–3000	5.64	1–10	1812	1000–10,000	162
Mixed	2.9	500	100–3000	25	10–100	850	100–1000	300
Fibrous	19.3	1200	100–3000	54	10–100	2200	1000–10,000	42.5
Lipid	13.0	15	5–200	18	10–100	207	100–1000	422
Calcium	0.3	5000	–	1270	–	–	–	–

## Recovery of material constitutive parameters

With simulated patient-specific target geometries at hand, the presented inverse method was used to recover the linear elastic and Yeoh material parameters. The solution space was bound to constrain the solution space to physically reasonable ranges within one order of magnitude, in line with the approaches taken in other inverse studies^23,29^, while allowing for substantial variation in the material parameters (Table 1). It was found through simulated uniaxial tensile tests that the nonlinear stress–strain response was sensitive to the Yeoh C_30_ parameter only at high strains (> 20%), which were not observed in the current patient-specific simulations. This rendered the objective function insensitive to the C_30_ parameters, which were therefore maintained at their assigned values during the recovery of Yeoh material parameters. Note however, that a three-parameter Yeoh model was maintained to avoid previously reported issues with non-convex energy contours associated with lower-order models^27^. Calcium parameters were also excluded from the recovery process of both material models due to its extreme sparsity in the present model. The inverse FE method was used to retrieve the remaining 4 and 8 parameters for the linear elastic and Yeoh constitutive models, respectively. During optimization to minimize the objective function ($\delta$), the base shape was iteratively simulated into the deformed shape using the current vector of assigned material parameters (Y^(*i*)^) for iteration *i*:

6 $$\Omega_{{\text{def}}}^{\left( i \right)} = \;\boldsymbol{\mathcal{S}}\left( {{\Omega_{{\text{base}}}},\Delta P,{{\mathbb{Y}}^{\left( i \right)}}} \right),$$

7 $${\delta^{\left( i \right)}} = \boldsymbol{\mathcal{D}}\left( {\Omega_{{\text{def}}}^{\left( i \right)},{\Omega_{{\text{target}}}}} \right),$$

8 $${{\mathbb{Y}}^*} = \;\arg \mathop {\min }\limits_{\mathbb{Y}} \delta .$$

where $\arg \mathop {\min }\limits_{\mathbb{Y}} \delta$ refers to the set of parameters ${\mathbb{Y}}$ that results in the minimum value of $\delta$. Since each NSGA-II run starts with a randomly generated initial population, 8 runs of the inverse FE method were conducted in each study to obtain a statistical overview of its performance. During each run, the NSGA-II algorithm used a population of 24 individuals propagated over 7 generations for recovery of linear material parameters. For nonlinear material parameter recovery, the number of generations was increased to 24 to ensure sufficient convergence to a Pareto optimal set of solutions with the increased number of parameters. The gradient-based NLPQLP algorithm was then invoked in both implementations for a maximum of 40 function evaluations with a termination residual error of 10^–9^.

## Noise sensitivity analysis

The simulated target shapes used in the earlier section provided an effective testbed for evaluating the accuracy of the proposed inverse FE method. However, such synthetic data represents an idealized version of the clinical equivalent, where acquisition noise, cardiopulmonary variations and motion, spatiotemporal inaccuracies, and meshing discrepancies may all influence data quality and subsequently influence the performance of the proposed method. Therefore, to study the effect of spurious noise and mimic real-world conditions, 5% and 20% random Gaussian noise were added to the displacement data used to generate ${\Omega_{{\text{target}}}}$ using linear elastic parameters:

9 $$U\; = \;{\Omega_{{\text{target}}}} - {\Omega_{{\text{base}}}}$$

10 $${U_{{\text{noisy}}}} = U\left( {1 + \boldsymbol{\mathcal{N}}\left( {0,n * \mathop {\max \left| u \right|}\limits_{u\; \in \;U} } \right)} \right)$$

11 $${\Omega_{{\text{target}} - {\text{noisy}}}} = {\Omega_{{\text{base}}}}\; + \;{U_{{\text{noisy}}}}$$

where each element of the original displacement vector (*U*) was scaled by a random sampling of a Gaussian distribution ($\mathcal{N}$) whose standard deviation is a percentage (*n*) of the maximum observed displacement in the model. Optimization was then carried out using the noisy target geometry (${\Omega_{{\text{target}} - {\text{noisy}}}}$) in a manner similar to the linear elastic case without noise.

## Method reproducibility

As a validation of versatility beyond the model employed in the aforementioned studies, the presented inverse method was used to recover nonlinear (Yeoh) material parameters in two additional patient-specific models. As evinced by the differing volume percentage of each tissue class (Supplementary Table S2), each model had varying degrees of material heterogeneity and phenotypes. Since the calcium volume percentages in these lesion models were greater than 1%, the C_10_ parameter for calcium was included in the recovery process for these cases, resulting in 9 recovered parameters.

## Results

### Recovery of material constitutive parameters

**Figure 4:**
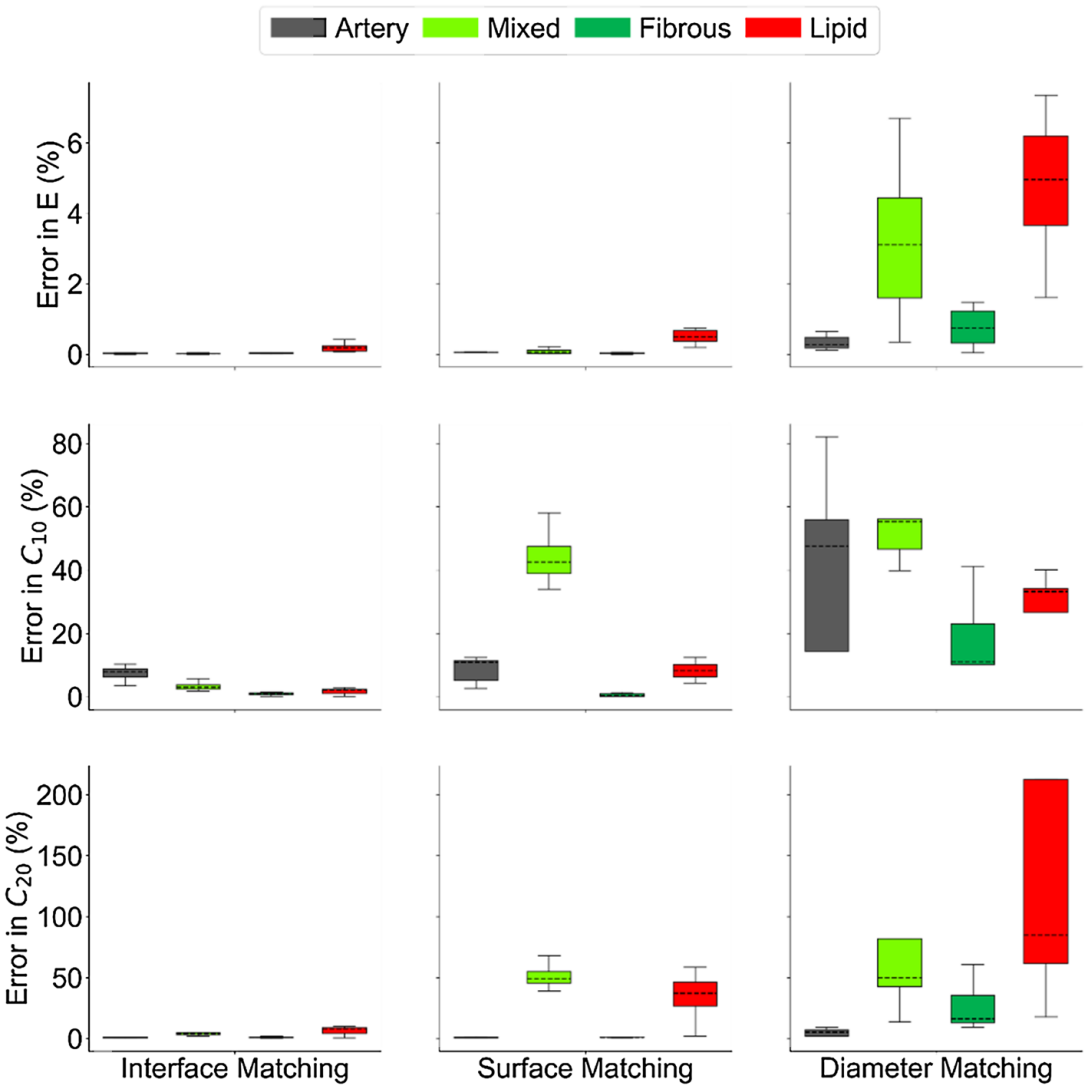
Errors in linear elastic (top row) and Yeoh (middle and bottom rows) material parameter recovery over 8 runs. The performance of the current interface matching method (left column) is compared to inner and outer surface matching alone (middle column), and diameter matching (right column). Full interface matching outperforms surface matching and diameter matching in all cases, but the distinction is more pronounced for the higher-order Yeoh model and for the predominantly intramural materials (mixed and lipid tissue). The median errors (dashed horizontal lines) were greater for material parameters with lower assigned value magnitudes. Boxes extend from 25th to 75th percentiles; range bars extend to extrema.

The inverse FE method accurately recovered linear elastic and Yeoh material parameters over 8 runs, with the optimal parameter vector (${{\mathbb{Y}}^*}$) determined by the optimization routine closely matching the vector of assigned parameters (${{\mathbb{Y}}_{{\text{assigned}}}}$) (Fig. 4). The 4 linear elastic parameters were recovered with errors of 0.1 ± 0.1%, while the 8 Yeoh material parameters were recovered with errors of 3.3 ± 3.0%, across 8 runs. The runs took 4.15 ± 0.63 h for the linear elastic case and 12.4 ± 1.1 h for the Yeoh material parameter recovery on 6 cores running at 2.8 GHz and with 24 GB RAM. For both cases, the parameters with the lowest assigned values (*E*_lipid_, $C_{20}^{{\text{lipid}}}$, and $C_{10}^{{\text{artery}}}$) exhibited the highest recovery errors (0.2 ± 0.1%, 6.6 ± 3.4%, and 7.3 ± 2.3%, respectively).

### Achieving superior performance by leveraging micro-morphological information

The stand-out feature of the presented method is its ability to recover multiple parameters for multiple materials through the incorporation of micro-morphological information in the form of intra-plaque tissue interfaces into the objective function, as evinced by the 8-parameter recovery (Fig. 4). To demonstrate the distinction of the method, its performance was directly compared to two approaches that do not take into account the micromorphology of the vessel wall, but instead use only macro-morphological information. In one approach, only the inner and outer vessel wall surfaces, instead of all intra-plaque interfaces, were considered in the objective function, in a manner inspired by Liu et al*.*^28^. In the other approach, the minimum and maximum diameters at a number of slices were compared, in a method similar to that used by Noble et al.^28^.

For the linear elastic case, the surface matching and diameter matching approaches were almost as effective as full interface matching, with recovery errors of 0.2 ± 0.4% and 2.2 ± 2.7%, respectively (Fig. 4). It is only when the number of recovered material parameters was increased to 8 that performance of the three approaches bifurcate, with errors of 18.6 ± 23.3% and 42.8 ± 45.4% for the surface and diameter matching approaches, respectively (Fig. 4). The difference in performance between these approaches and the one presented here was especially pronounced for the mixed and lipid tissue types (Fig. 4). One plausible explanation is that both fibrous and artery tissue have a significant overlap of nodes with the inner and outer surfaces of the vessel wall, while the mixed and lipid tissue are confined mostly to the interior of the vessel. This indicates that while the surface-matching method, and to an extent the diameter matching method, can adequately capture the mechanical behavior of tissues which are adjacent to the matched inner or outer surfaces and sufficiently prevalent, they are insensitive to tissues which are predominantly intramural or of low prevalence. These findings highlight the benefit of leveraging micro-morphological information in defining the objective function to achieve superior mechanical characterization performance.

### Noise sensitivity analysis

**Figure 5:**
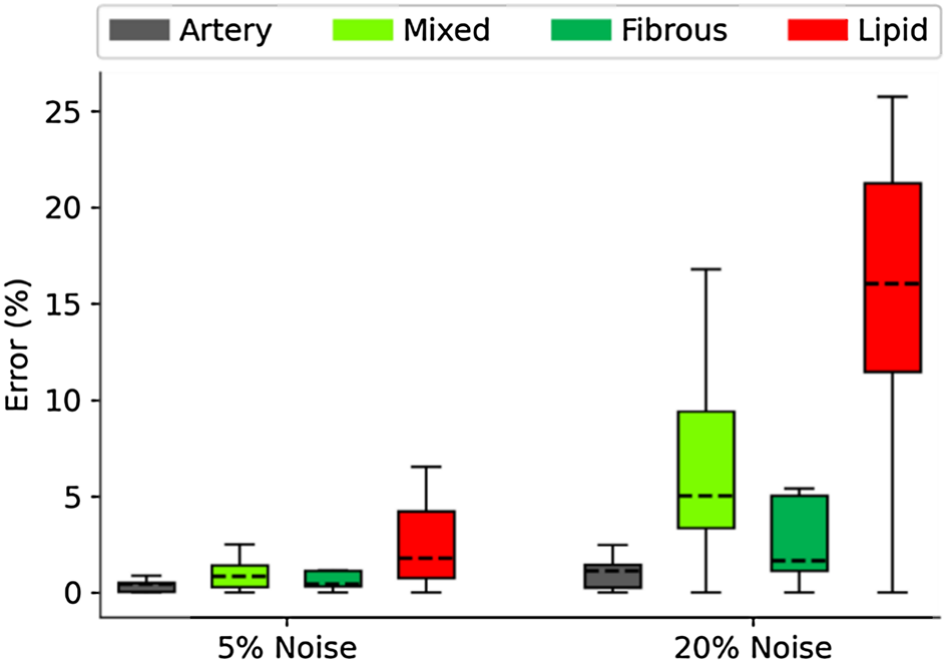
Errors in linear elastic parameter recovery when 5% and 20% Gaussian noise were imposed to generate noisy target geometry.

Figure 5 shows the errors in linear elastic parameter estimation upon application of random Gaussian noise to the target shape. The errors for the material parameters were 1.3 ± 1.6% for the 5% noise addition. For the 20% Gaussian noise case, arterial tissue, mixed tissue and fibrous tissue were all recovered with errors of 4.2 ± 4.3%; the softest material, lipid, was recovered with errors of 20.6 ± 13.9%. For both noise levels, lipid tissue showed the greatest errors in parameter recovery (2.9 ± 2.4% and 20.6 ± 13.9% for 5% and 20% noise, respectively), followed by mixed tissue (1.1 ± 0.8% and 7.5 ± 5.1% for 5% and 20% noise, respectively)—the second softest tissue type.

In addition to the noise inherent to the image-based geometry, in vivo application of the method will also be susceptible to noise and uncertainty in the corresponding pressure readings. The impact of such pressure perturbations on material property recovery was also studied by perturbing the lumen pressure used to generate target geometry by ± 5% and ± 10%. The results (Supplementary Table S1) were as expected, with the errors in linear elastic stiffness being approximately equal to that of the applied perturbation.

### Method reproducibility

Reproducible, accurate performance was demonstrated above through repeated material recovery in a single patient (Patient A; Supplementary Table S2). Generalizability of the performance was demonstrated by conducting single proof-of-concept runs of the inverse FE method for two other patient-specific models (Patients B and C; Supplementary Table S2). The material parameters (Table 2) were recovered with errors of 3.0 ± 4.7% across both models, with C_20_ for mixed tissue (Patient B) and C_20_ for lipid tissue (Patient C) having the highest errors (11% and 17.4%, respectively).

**Table 2:** Final errors in Yeoh material parameter recovery during a single run for Patients B and C.

Material	Artery		Mixed		Fibrous		Lipid		Calcium
Parameter	C_10_	C_20_	C_10_	C_20_	C_10_	C_20_	C_10_	C_20_	C_10_
Ground truth (kPa)	5.6	1812	25.0	850	54.0	2200	18.0	207	1270
**Error (%)**									
Patient B	5.3	0.9	7.9	11.0	0.4	0.2	1.3	2.1	1.7
Patient C	1.4	0.4	0.1	0.3	0.2	0.2	3.9	17.4	0.1

## Discussion

The successful recovery of 8 Yeoh material parameters to within 10% of their true values over 8 runs has emphatically demonstrated the potential of the presented inverse FE method for in vivo multi-parameter, multi-material characterization. By recovering nearly all linear elastic parameters with median errors of < 5% under the application of noise, the method also offers confidence in its ability to withstand the challenges of real-world conditions.

To evaluate the clinical viability of the method, we benchmark it against the ideal characterization method, which would preferably: (i) use a minimal number of pullbacks; (ii) utilize a 3D reconstruction of the diseased vessel; (iii) be capable of recovering multiple materials; and (iv) incorporate nonlinear material models with multiple parameters.

One of the drawbacks of our proposed inverse method is the need for more than a single pullback, with the number of pullback acquisitions limited by constraints imposed by clinical care routines, such as patient risk and costs. Considering the added benefit of being able to characterize tissue types and hence better inform clinical interventions, we believe that extra pullbacks may be justified in some cases, as they are for cases involving long lesions, though further cost–benefit analysis is warranted. With this in mind, the presented method is more viable than previous approaches in a clinical setting as it requires only two pullbacks to recover an increased number of material parameters.

While 2D slices can be used to recover material properties, there are important facets of the physiological environment that are unaccounted for in such representations, the most obvious of which are out-of-plane stresses and loads. By using 3D FE models, our method not only captures 3D morphological information, but also ensures that the applied loads are physiologically representative.

Clinically-relevant atherosclerosis is commonly marked by complex lesions with heterogeneous distribution of a diverse set of tissue elements. Hence, accounting for material heterogeneity and achieving multi-material recovery is paramount in understanding the state of the disease. In this regard, our method has shown the state-of-the-art ability to delineate and recover the material parameters of 5 different tissue classes.

Finally, as expounded in the introduction, in silico simulations are increasingly using sophisticated nonlinear material models to better capture the physiological biomechanical response of arterial tissue. While our study was conducted with an isotropic hyperelastic model for reasons of computational efficiency, more physiologically adherent simulations would incorporate anisotropic models that consider the fiber orientations within the arterial wall as well^40^. Considering that these complex models tend to have more material parameters, the importance of the multi-parameter recovery conducted in this study is apparent.

Apart from being able to satisfy the criteria for clinical viability laid out above, our method also carries other advantages. The reliance on 3D morphological information obviates the need for additional image processing including recovery of local strain state (as in elastography imaging). This not only circumvents common sources of error, but also makes the approach readily applicable across imaging modalities, some of which may not possess an elastography module or developed capabilities. Additionally, by using a surface comparison that does not require node-to-node correspondence, the method reduces the meshing pre-processing constraints. This also means that the simulated mesh can be fine-tuned to optimize for performance and accuracy without requiring corresponding manipulation of the target mesh to maintain parity. Lastly, it should be noted that our results indicate that the use of only two displacement states (*baseline* and *target*) acquired at physiological extrema (peak systole vs. late diastole) is sufficient to fully recover underlying constitutive tissue properties. As such, a final advantage of our method is the avoidance of any *external* intravascular pressure elevation, where instead full material recovery is permitted simply using physiological pressure variations observed during normal homeostatic cardiovascular performance. Enhanced mean arterial pressures could possibly be achieved by means of intravascular contrast agents^35,41^, or pharmaceutical vasopressors^42^, however, its utility in combination with our approach would require further future attention.

Although the presented inverse FE method provides numerous advantages over the existing state of the art, it comes with its own set of limitations. While it requires that the pressure state within the vessel be known, pressure at the point of image acquisition is not commonly recorded in clinical practice. This presents a current translational gap since in this study, pressure data were not available and therefore assigned. However, ongoing progress in imaging hardware is likely to make this information increasingly available and accessible for use in the clinic.

Another limitation of the method as implemented here is that a homogeneous pressure is distributed along the length of the lumen of a *supposedly stress-free* base geometry configuration. In current clinical practice, arterial pressures vary throughout acquisition sequences, such that varying luminal pressures and intramural pre-stresses need to be represented. The validity of the stress-free assumption is especially compromised when using nonlinear hyperelastic models which better approximate the behavior of biological tissue. Fortunately, there exist computational approaches to recover the zero-pressure geometry of a vessel if the imaged pressure state is known^43–45^. Deformed geometry can thereafter be simulated from the unloaded (zero-pressure) state using a spatially varying luminal pressure to reflect the fluctuating vascular states captured during imaging. These existing modules can be integrated with the objective function definition and associated methods introduced in this paper to implement these features for in vivo application. Likewise, incorporation of higher-order anisotropic material models was not explored in this work. However, these material models can easily be combined with the interface-matching inverse methodology presented here. Albeit both representing valuable additions worthy of continued investigation, the incorporation of any of the above was however deemed beyond the scope if this initial work, in which instead we have intended to map out possibilities of inverse material characterization using a novel interface-matching approach.

The incorporation of micro-morphological information into the objective function also serves as a limitation in terms of the modalities to which the method can be applied and its dependence on the accuracy of the plaque annotation that precedes the FE mesh generation. Each tissue class is also assumed to be internally homogeneous, which need not always be the case. Still this approach provides among the most granular and sophisticated tissue characterization of its kind.

Finally, the studies reported herein demonstrate the efficacy and robustness of the approach through in silico experiments. While the noise sensitivity analysis mitigates concerns of clinical viability to a certain extent, the ultimate challenge is to reproduce the results in a physical experimental or clinical setting. In this light, a preliminary study using the presented method to characterize homogenous phantoms has been carried out^46^. Future work is envisioned to experimentally validate the method through recovery of material properties of artery-mimicking phantoms as well as in pre-clinical settings. In this vein, recovering the properties of porcine tissue in a living animal model may provide compelling evidence in support of the method’s in vivo viability, although such tissue is known to present less heterogeneity than human tissue. While the present in silico validation benefited from known, exact ground truth values of constitutive properties, a thorough validation of future physical testing will also require excised tissue to be tested ex vivo.

## Conclusion

This work presented a novel inverse FE method that makes use of the heterogeneity inherent in diseased coronary artery tissue to accurately and robustly recover nonlinear material parameters of multiple plaque components. It scaffolds a framework for future clinical application where lesion-specific mechanical characterization can help better inform clinical scientists and interventionalists of the potential impact of certain morphological phenotypes on different clinical interventions. In allowing for the recovery of more accurate and physiologically relevant material parameters, it can also facilitate high-fidelity computational studies which are imperative for evaluating the lesion-dependent impact of novel and legacy interventional devices and processes. While practical challenges must still be resolved prior to clinical application, the unparalleled feasibility of the presented method sets into motion the progressive technical advancements which may realize tangible benefits for the care of patients with cardiovascular disease.

## Supplementary Information

Supplementary Information.

## Data Availability

The datasets generated and/or analyzed during the current study are not publicly available due to restrictions imposed on distribution of clinical data used in this work, but are available from the corresponding author on reasonable request.
