# Supplementary material for: An inverse method for mechanical characterization of heterogeneous diseased arteries using intravascular imaging
Source: Sci Rep. 2021 Nov 18;11:22540. doi: 10.1038/s41598-021-01874-3 (PMC8602310; doi:10.1038/s41598-021-01874-3)
Supplement: Supplementary file 1 — Supplementary Information. [file 41598_2021_1874_MOESM1_ESM.docx]

**An Inverse Method for Mechanical Characterization of Heterogeneous Diseased Arteries using Intravascular Imaging**

***Supplementary Information***

**Effect of pressure perturbations on linear elastic parameter recovery**

The effects of pressure perturbations on the recovery process were studied by adding a pressure perturbation during the generation of target geometry ($\Omega_{\mathrm{target}}$) prior to the inverse process. Overall, the errors in parameter estimation scaled approximately linearly with the induced lumen pressure perturbations. As expected, a reduction in applied lumen pressure results in a lower Young’s modulus across all the materials, and vice versa.

| **Pressure perturbation (%)** | **Error in linear elastic parameter recovery (%)** | | | |
| --- | --- | --- | --- | --- |
|  | **Artery** | **Mixed** | **Fibrous** | **Lipid** |
| -5 | -5.0 | -5.3 | -5.0 | -4.6 |
| 5 | 5.0 | 5.3 | 5.0 | 5.7 |
| -10 | -10.0 | -10.3 | -10.0 | -10.1 |
| 10 | 10.0 | 10.1 | 10.1 | 10.7 |

**Supplementary Table S1.** Errors in Young’s modulus recovery for ±5% and ±10% perturbation in applied lumen pressure during the generation of $\Omega_{\mathrm{target}}$.

**Patient models used for *in silico* verification and generalizability studies**

In total, three different patient-specific FE models were used in this study. The sensitivity of the method to mesh density was first ascertained using the images obtained from Patient A. Across an order of magnitude difference in number of elements (66,780 to 534240), the method was found to be insensitive to mesh density during linear elastic recovery, with errors within 1%. For computational efficiency, all subsequent studies were conducted with coarser meshes.
Data from Patient A (Table 1) was used for the verification process and the noise sensitivity studies. Models generated from Patients B and C (Table S2) were used as a proof of the applicability of the method in different lesion phenotypes.

| **Patient** | **Segment Length (mm)** | **Model Elements** | **Relative Volume (%)** | | | | |
| --- | --- | --- | --- | --- | --- | --- | --- |
|  |  |  | **Artery** | **Mixed** | **Fibrous** | **Lipid** | **Calcium** |
| A | 19.7 | 66,780 | 64.6 | 2.9 | 19.3 | 13.0 | 0.3 |
| B | 3.8 | 40,091 | 60.1 | 3.6 | 19.2 | 15.7 | 1.4 |
| C | 8.5 | 40,236 | 87.9 | 0.9 | 7.9 | 1.9 | 1.5 |

**Supplementary Table S2.** Characteristics of the various patient-specific lesion models used to demonstrate non-linear parameter recovery performance stability across clinical use cases.
